# Confirmation Bias in Studies of Nestmate Recognition: A Cautionary Note for Research into the Behaviour of Animals

**DOI:** 10.1371/journal.pone.0053548

**Published:** 2013-01-23

**Authors:** Ellen van Wilgenburg, Mark A. Elgar

**Affiliations:** Department of Zoology, University of Melbourne, Melbourne, Victoria, Australia; Stanford University, United States of America

## Abstract

Confirmation bias is a tendency of people to interpret information in a way that confirms their expectations. A long recognized phenomenon in human psychology, confirmation bias can distort the results of a study and thus reduce its reliability. While confirmation bias can be avoided by conducting studies blind to treatment groups, this practice is not always used. Surprisingly, this is true of research in animal behaviour, and the extent to which confirmation bias influences research outcomes in this field is rarely investigated. Here we conducted a meta-analysis, using studies on nestmate recognition in ants, to compare the outcomes of studies that were conducted blind with those that were not. Nestmate recognition studies typically perform intra- and inter colony aggression assays, with the *a priori* expectation that there should be little or no aggression among nestmates. Aggressive interactions between ants can include subtle behaviours such as mandible flaring and recoil, which can be hard to quantify, making these types of assays prone to confirmation bias. Our survey revealed that only 29% of our sample of 79 studies were conducted blind. These studies were more likely to report aggression among nestmates if they were conducted blind (73%) than if they were not (21%). Moreover, we found that the effect size between nestmate and non-nestmate treatment means is significantly lower in experiments conducted blind than those in which colony identity is known (1.38 versus 2.76). We discuss the implications of the impact of confirmation bias for research that attempts to obtain quantitative synthesises of data from different studies.

## Introduction

“…for it is a habit of mankind to entrust to careless hope what they long for, and to use sovereign reason to thrust aside what they do not desire.” From *History of the Peloponnesian War* 431 B.C., Thucydides

Confirmation bias, a well-documented phenomenon in psychology, is the tendency of humans to seek out evidence and interpret it in a manner that confirms their existing ideas and hypotheses [1]–[7]. Confirmation bias is often described as a result of automatic processing, occurring more or less unintentionally but nevertheless potentially distorting the data collected in scientific research. Ideally, scientific researchers avoid confirmation bias by searching for falsifying, as well as confirming evidence [8], [9]. However, in reality, scientists often have high stakes for obtaining particular research outcomes [10], and the expectations for an experiment can potentially affect which data are collected and how they are interpreted and reported [1], [2]. For example, two-thirds of recording errors in several psychological studies were biased in the direction of the observer's hypothesis [1]. The extent to which observation bias influences the outcomes of a study will depend on the kind of observations that are being made [1], [7], [11]. Observations are more prone to bias if 1) the variable is not clearly defined, 2) the subject under observation is hard to perceive, 3) the observations require subjective assessment, and 4) the observer has an interest in the outcome of the study.

Confirmation bias can be avoided by designing experiments in which the observers are blind to the treatment assignment of their subjects [12]–[18]. For example, to test whether consumers have a taste preference for one brand of pop over another, the identity of the pop should be concealed because otherwise the subjects tend to prefer the brand with which they are more familiar. Nowadays, blind experiments are commonplace in many scientific disciplines, including pharmacology, market research, psychology, physics and certain branches of biology. Indeed, in some fields of research, blinding of experiments is essential for publication.

Such an experimental tradition appears to be less widely adopted in the field of animal behaviour, where researchers collect observational data that may be subject to systematic error. For example, a survey by Gamboa et al. [18] revealed that only 27% of 33 studies that investigated kin-recognition and were published in the journal *Animal Behaviour* between 1987 and 1989 mentioned blind assays. Studies of animal behaviour may be particularly prone to confirmation bias, especially when a certain degree of interpretation is required— typically when the behaviours are rapid, subtle or similar in appearance to other behaviours. Yet remarkably few studies have investigated the extent to which confirmation bias influences research outcomes in animal behaviour [19]–[21]. Almost half a century ago Cordaro and Ison [21] conducted an experiment in which they asked students to observe the behaviour of planaria (non-parasitic Turbellaria flatworms). One group of observers were told the planaria would move and turn frequently, whereas the other group of observers were told their planaria rarely move and turn. In reality, the planaria were randomly allocated to the two groups. The group of students anticipating high-activity animals found that the planaria moved on average 18 times and turned 49 times, while the group of students anticipated low-activity animals reported the planaria moved on average once and tuned 10 times. Similar studies by Rosenthal and Fode [19] and Marsch and Hanlon [20] conducted on the behaviour of rats and salamanders respectively, also report that *a priori* expectations can bias behavioural observations. However, it has to be noted that the observers in all three studies were undergraduates, who generally have little or no training in conducting behavioural observations. More experienced researchers may make fewer observational errors and their data may therefore be more reliable.

An alternative approach to addressing the question of whether confirmation bias affects research into animal behaviour is to compare the outcomes of published studies that are conducted blind with those that are not. If behavioural observations are influenced by confirmation bias, then the outcomes of studies that have been conducted blind should have smaller effect sizes than similar experiments that were not conducted blind.

In this study, we explore the evidence for confirmation bias in studies of animal behaviour by focussing on a single research topic—nestmate recognition in ants. Our intention is to use studies of nest-mate recognition as a ‘model system’ to highlight the potential impact of confirmation bias, which is a potential issue for all quantitative research, including animal behaviour. Ants, like other social insects, maintain colony cohesion by recognizing and, if necessary, discriminating against conspecifics that are not members of their colony [22]–[26]. The mechanisms of nestmate recognition have received considerable attention during the past 25 years [27]–[29]. Aggression assays are useful tools when trying to test hypotheses regarding the influence of context, environment and heritability on nestmate recognition. Experimenters have used a variety of methods testing inter-colony aggression ranging from one-on-one assays to group encounters, which often yield similar results [30]. However, like any other observational method of data acquisition, aggression assays have their limitations and great care must be taken to avoid false positive and negative results when designing nestmate recognition studies [31]. Several authors stress the importance of conducting these types of assays blind to the treatment [18], [31]. Nestmate recognition experiments typically involve intra- and inter colony aggression assays with the *a priori* expectation that there should be little or no aggression among nestmates. Since little or no aggression is expected among nestmates, we expect aggression to be less frequently reported in trials involving nestmates that are not conducted blind, compared with those conducted blind – that is, the experimenter has no knowledge of whether the ants involved in the assay comprise nestmates only, or a mixture of nestmates and non-nestmates.

Studies of nestmate recognition are particularly suitable to investigate confirmation bias for a variety of reasons. First, many studies use similar experimental designs, allowing relatively straightforward comparisons across studies. Second, aggressive behaviour in ants can include subtle behaviours such as mandible flaring and recoil that are hard to quantify, making the assays potentially prone to confirmation bias. Using a meta-analysis, we specifically ask 1) what proportion of studies of nestmate recognition are conducted blind, and 2) do the outcomes of blind studies differ from those of non-blind studies?

## Methods

We searched for papers on nestmate recognition in ants using *ISI Web of Science* (Thomson Reuters) search engine, with the search terms “nestmate recognition” or “nest mate recognition”. We conducted the last search in July 2011. To be included in our analyses, studies must have conducted a nestmate recognition experiment on ants that included both a nestmate (control) and non-nestmate aggression assay. Aggression assays had to involve either live, chilled or dead ants. To determine whether a study was conducted blind or not (e.g. whether the observers of the assays were aware of the colony identity of the workers) we carefully read through the method section of each paper. We deemed a study as blind only if this was explicitly stated, and categorised the remaining studies as non-blind. It is possible that some of the studies we deemed non-blind were, in fact, conducted blind. However, the alternative of contacting the authors of all papers to ascertain whether their study was conducted blind or not introduces several sources of bias that we could not control. For example, authors that did not explicitly state their study was conducted blind may be less likely to recall whether the study was done blind or more likely to remember incorrectly.

We treated different experiments included in the same publication, different studies by the same author, and different studies on the same species as independent because leaving them out may lead to greater loss of information and distortion of the results than those caused by their potential non-independence [32]. The studies included in our sample are listed in Table 1. We do not include details of the experimental methods for each study because we see no value in drawing attention to the methods of individual studies. The types of aggression assay and the methods of scoring vary between studies. For example, assays may involve one-on-one encounters in a petri-dish [33], or placing ants into a nest [34] or foraging trail [35]. Aggression may then be scored as simply the presence or absence of aggression [36] or it may be scored on a scale based on specific behaviours thought to represent increasing aggression [35]. A meta-analysis requires a certain level of homogeneity among studies, limiting the number of studies that can be included in the analysis. Since our dataset is so varied, a single type of analysis would necessarily exclude many studies. We therefore chose to analyse our data using two different methods that differ in their selection criteria, resulting in two mostly overlapping, but nonetheless different samples. First, we tested whether there was any difference in the frequency with which aggression was reported in nestmate trials in blind versus non-blind studies. Second, we compared the nestmate vs non-nestmate effect size between blind and non-blind experiments.

**Table 1:** Evidence for aggression among nestmates in studies of nestmate recognition in ants.

Taxa	Aggression among nestmates?	d	Var (d)	Reference
Paraponerinae				
*Paraponera clavata*	Yes			[46]
Ponerinae				
*Odontomachus Bauri*	No			[47]
*Pachycondyla inversa*		0.840	0.076	[48]
*Pachycondyla subversa*		1.160	0.147	[48]
*Pachycondyla villosa*		0.762	0.075	[48]
Myrmeciinae				
*Myrmecia nigriceps*	Yes			[40]
Pseudomyrmecinae				
*Pseudomyrmex ferruginea*	No			[49]
*Pseudomyrmex pallidus*		1.248	0.154	[50]
Dolichoderinae				
*Iridomyrmex purpureus*		3.629	0.157	[35]
*Iridomyrmex purpureus*	No			[51]
*Iridomyrmex purpureus*	Yes			[52]
*Iridomyrmex purpureus*	Yes	0.955	0.045	[53]
*Linepithema humile*	No			[54]
*Linepithema humile*				[55]
*Linepithema humile*		9.678	1.321	[56]
*Linepithema humile*		1.954	0.006	[57]
*Linepithema humile*	No	0.225	0.027	[58]
Ectatomminae				
*Ectatomma ruidum*	No			[59]
*Ectatomma ruidum*	Yes			[60]
*Ectatomma tuberculatum*				[61]
Formicinae				
*Anoplolepis gracilipes*	No			[62]
*Camponotus aethiops*		1.525	0.047	[63]
*Camponotus aethiops*		1.059	0.095	[64]
*Camponotus aethiops*	No	3.069	0.109	[65]
*Camponotus atriceps*		0.939	0.185	[66]
*Camponotus cruentatus*				[67]
*Camponotus fellah*	No	1.512	0.205	[68]
*Camponotus fellah*	No			[69]
*Camponotus fellah*	No			[70]
*Camponotus floridanus*	No			[71]
*Camponotus floridanus*	No			[72]
*Camponotus japonicus*	Yes	1.384	0.113	[73]
*Camponotus rufifemur (black)*	Yes			[25]
*Camponotus rufifemur (red)*	Yes			[25]
*Camponotus rufipes*	No	3.484	0.420	[74]
*Camponotus vagus*	No			[75]
*Camponotus yamaokai*	No			[76]
*Cataglyphis cursor*	No			[77]
*Cataglyphis cursor*	No	2.280	0.114	[78]
*Cataglyphis iberica*	No			[79]
*Cataglyphis niger*		4.343	0.480	[80]
*Formica exsecta*	Yes	0.139	0.083	[81]
*Formica exsecta*	No			[82]
*Formica exsecta*	No			[83]
*Formica japonica*	No			[84]
*Formica montana*	Yes			[85]
*Formica pratensis*	No			[86]
*Formica pratensis*	Yes	1.213	0.064	[87]
*Formica rufibarbis*		1.309	0.130	[88]
*Formica selysi*	Yes			[89]
*Lasius neglectus*	No			[90]
*Oecophylla smaragdina*				[91]
*Plagiolepis pygmaea*	No	7.295	0.064	[92]
Myrmicinae				
*Acromyrmex lobicornis*	Yes			[93]
*Acromyrmex subterraneus*	No			[94]
*Aphaenogaster senilis*		1.676	0.025	[95]
*Aphaenogaster senilis*	No			[96]
*Apterostigma collare*	No			[97]
*Cataulacus mckeyi*	No			[98]
*Leptothorax ambiguous*	No			[99]
*Leptothorax longispinosus*	Yes			[100]
*Leptothorax longispinosus*		4.951	0.033	[101]
*Leptothorax retractus*	No			[102]
*Leptothorax sp B*	Yes	1.661	0.168	[102]
*Monomorium pharaonis*				[103]
*Myrmica rubra*		0.506	0.145	[104]
*Myrmica rubra*	Yes	1.694	0.165	[105]
*Pheidole megacephala*	No			[106]
*S. invicta, S. richteri hybrids*	No			[107]
*Solenopsis invicta*	Yes	3.938	0.235	[108]
*Solenopsis invicta*	No			[107]
*Solenopsis invicta*		4.971	0.090	[109]
*Solenopsis invicta*	No			[110]
*Solenopsis invicta*		3.064	0.084	[111]
*Solenopsis richteri*	No			[107]
*Temnothorax crassispinus*	Yes	0.370	0.020	[112]
*Temnothorax nylanderi*	Yes	0.183	0.020	[112]
*Temnothorax unifasciatus*	Yes	−0.174	0.020	[112]
*Tetramorium bicarinatum*	No			[113]

The table gives the effect sizes measured as Hedge's d and the variance of Hedge's d (Var(d)). (A full table, including assignment of experimental protocol, is available on request to the authors.)

### Frequency of aggression reported in nestmate trials

We compared the frequency with which aggression is reported in blind *versus* non-blind studies by searching for papers that specifically mention the presence or absence of aggression in nestmate trials. To be included in this analysis studies had to either present their data as presence/absence of aggression, or explicitly mention that aggression was or was not found in the nestmate trials.

### Differences in effect sizes of aggressive behavior

Studies included in our analysis of effect sizes had to report descriptive statistics (means, standard deviation or standard error and sample size) of a measure of aggression for both nestmate and non-nestmate trials. We retrieved this information either directly from the text or estimated it from the figures, and converted standard errors to standard deviations.

### Statistical methods

We used Fisher's exact test (with each experiment as an independent value) to reveal whether aggression was more frequently reported in nestmate trials that were conducted non-blind than blind. We compared the effect size of blind and non blind studies using MetaWin 2.0 [37]. We measured the effect using Hedges d [38], which provides a standardized mean difference between nestmate and non-nestmate mean value of the aggression assay. If a study included several different non-nestmate treatments (for example non-nestmates of colonies from different distances) we calculated the average level of aggression and standard deviations over these treatments.

We compared the effect sizes across studies using mixed effect models that allow for fixed differences between groups of studies (in our case blind versus non-blind), and assume that differences among studies within a class are due to both sampling error and random variation [37], [39]. Although the statistical power of mixed models is lower than that of fixed-effects models, the assumptions of mixed models are much more likely to be met in most meta-analyses in ecology [37], [38]. We report the mean response ratios and their 95% confidence limits. For comparisons between blind and non-blind studies we examined the between-group heterogeneity using a Chi-square test, Q_B_ [38].

### Potential sources of bias

We assessed potential bias both within individual studies included in the meta-analysis and across the meta-analysis as a whole, considering studies excluded for any reason including failure to publish non-significant results. We examined individual studies for any potential sources of bias and did not find any experimental design limitations other than the absence of blinding in the non-blind studies, so there is no evidence for bias within studies that would affect our conclusions. At the level of the whole meta-analysis, publication bias could influence our results if statistically non-significant results are under-reported. This would only affect our conclusions if blind studies were less likely to find significant results than non-blind studies and a greater proportion of blind studies remained unpublished for this reason. While there are tests for publication bias [37], we were not able to test for statistically significant differences in publication bias between blind and non-blind studies, particularly since such tests would require much larger sample sizes than are available with the current published literature. So while it seems unlikely that a difference in publication bias would influence our main conclusions, it is possible and should be considered when interpreting our results.

## Results

**Figure 1:**
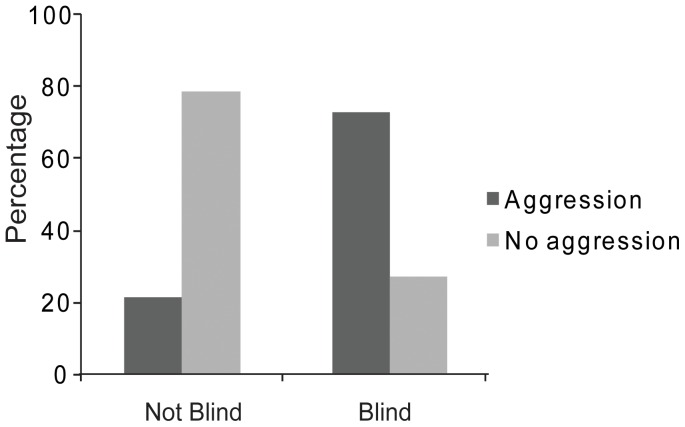
Percentage of non-blind and blind studies reporting aggression in control trials.

**Figure 2:**
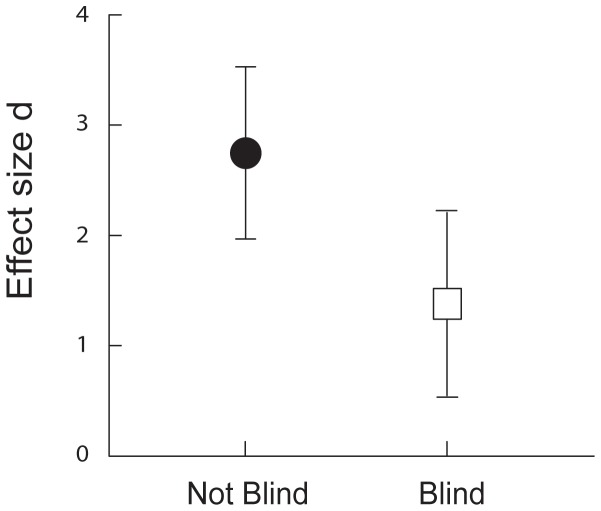
Mean effect size (d) and 95% CI of non-blind and blind studies.

We obtained published reports of 156 nestmate recognition experiments in ants. 79 of these studies involved live, chilled or dead ants and included nestmate controls. In 29% (23 of 79) of these studies, the experiments were conducted blind with respect to colony identity. Six of the studies that were not conducted blind according to colony identity were nonetheless blinded for different treatments among non-nestmate aggression assays (for example genetic distance). We could infer whether aggression was reported for the nestmate controls in 57 of the 79 experiments. We found that blind experiments were significantly more likely to report aggression in the controls than those not conducted blind (11 out of 15, or 73% versus 9 out of 42 or 21%, *P*<0.001, Figure 1). Thirty-three experiments fulfilled our inclusion criteria for the meta-analysis (see Methods and Table 1) and 15 (45%) of these were conducted blind. Blind experiments yielded a significantly lower treatment effect than non-blind experiments (Blind: 0.46≤1.38≤2.29; Non-Blind: 1.92≤2.76≤3.83; Q_B_ = 5.61, *P* = 0.018, Figure 2).

## Discussion

Our meta-analysis provides evidence of confirmation bias in studies of nestmate recognition in ants. Experimental aggression assays that investigate nestmate recognition in ants can be conducted blind with respect to the origin of ants. Aggression among nestmates was three times more likely to be reported in blinded than non-blinded experiments. Furthermore, the effect size – the differences between the level of aggression among nestmates and that among non-nestmates – in non-blind experiments was twice that of blind experiments. Remarkably, less than a third of the studies in our sample were conducted blind. While it is possible that some of the studies in our sample were incorrectly labelled non-blind, such incorrect allocations act against the observed pattern that blinded experimental designs are typically reported. Further, the minimum number of incorrectly assigned studies required to render our initial analysis non-significant would be 7, or 12% of the studies included in our sample, which seems an unrealistically high error rate.

There may be several explanations for the magnitude of confirmation bias in the non-blind studies. First, ants are relatively small, fast moving, and their aggressive behaviour may be particularly hard to quantify. While nestmates usually behave amicably, handling of worker ants may occasionally elicit aggression among nestmates and a rapid bite to the leg or a mandible flare can be easily overlooked if such behaviour is not expected. Moreover, aggressive behaviours in ants may appear similar to other non-aggressive behaviours. For example, ants may flare their mandibles as a threat to intruders, but may also open their mandibles in order to solicit trophyllaxis. The more frequently the behaviour requires interpretation, the more likely the data become prejudiced. The dramatic effects of confirmation bias revealed in our analysis highlight the impact of automatic, unintentional processing, even when the experiments are typically utilised to address broader questions, and the magnitude of the difference in the behaviour of nestmates and non-nestmates is often of little consequence. Indeed, reporting an absence of aggression in both nestmate and non-nestmate trials is more remarkable [40].

Our analysis raises the question of whether the degree of confirmation bias revealed in this study reflects research in animal behaviour more generally. While confirmation bias is an issue for almost all kinds of quantitative research, there is likely to be considerable variation across (and within disciplines) in the degree to which it is controlled. Several factors may influence this variation. Most obvious is the degree of prior expectations, which may derive from a compelling theoretical framework and/or empirical evidence – both of which are true for studies of nestmate recognition in ants. On that basis, we might expect similar levels of confirmation bias in, for example, studies of winner and loser effects [41], [42] or those that investigate the relationship between predator vigilance and group size in vertebrates [43]. Second, the accuracy of observations may be important: as already noted, behavioural observations on ant aggression may be prone to bias because ants are relatively small and fast moving, and so aggression may be difficult to discern accurately. Finally, observation biases may be more prevalent in studies of animals that have humanlike behaviours [44]. If so, the level of confirmation bias described here may be at the lower end of the spectrum.

Less than a third of the studies in our sample were conducted blind, a statistic similar to that published over 20 years ago for this kind of research [18]. This is surprising, since confirmation bias is widely documented, and textbooks on scientific methods and experimental design encourage blind experimentation [12]–[18]. While the nature of some experiments or sampling observations in animal behaviour would make it technically impossible to conduct them blind, there may be other explanations why blinding is so rare. Some researchers may choose to conduct open trials in the belief that the behaviour in question is easy to classify and therefore not prone to bias. Such a view is most likely mistaken, as confirmation bias occurs more or less unintentionally and scientists generally do not distort data intentionally [45]. Observations of the behaviour of animals are often thought to be less subjective than, for example, the qualitative observations in human psychology or market research. Our data suggest that, again, this view may be mistaken – in the absence of data such as that provided here, researchers may underestimate the extent to which confirmation bias can influence the outcomes of a study. Unfortunately, being informed about confirmation bias may not solve the problem entirely: around 75% of studies in special education research, in which the role of confirmation bias had been extensively investigated, made no precaution against it [11]. Finally, open trials may be preferred simply because of the additional costs of conducting experiments blind. These costs may not be trivial because blinding typically requires a second person to label the treatments. It is likely that the most effective way of encouraging researchers to conduct experiments blind is if journals set a benchmark for experimental design. If there is a trade-off between the chance of error and productivity, it may be otherwise unrealistic to expect researchers to utilise methodological standards beyond what is expected by scientific research journals.

The results for most studies that were not conducted blind are likely robust because the treatment effect size in both blind and non-blind studies is much greater than the difference in the effect sizes. Nevertheless, the prevalence of confirmation bias in studies that are not conducted blind has significant implications for synthetic research that relies on published data, such as comparative or meta-analyses. For example, an inter-specific analysis of the variation in the level of aggression expressed toward non-nestmates may yield a distorted pattern if the experimental methodology is linked to particular taxonomic groups. As a precaution, such studies may be advised to include only blind studies in these types of research. We hope that our analysis will stimulate renewed interest in designing experiments in a way that bias is minimized and set a methodological benchmark for research in animal behaviour more generally.

## Supporting Information

Checklist S1 **PRISMA 2009 Checklist.**
(DOC)

Flow Diagram S1 **PRISMA 2009 Flow Diagram.**
(DOC)
