# Supplementary material for: Confirmation Bias in Studies of Nestmate Recognition: A Cautionary Note for Research into the Behaviour of Animals
Source: PLoS One. 2013 Jan 23;8(1):e53548. doi: 10.1371/journal.pone.0053548 (PMC3553103; doi:10.1371/journal.pone.0053548)
Supplement: Flow Diagram S1 — PRISMA 2009 Flow Diagram. (DOC) [file pone.0053548.s002.doc]

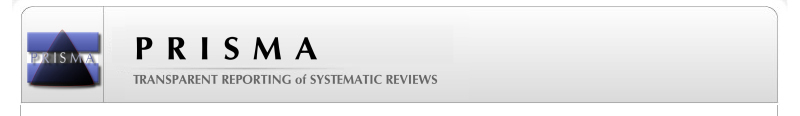
**PRISMA 2009 Flow Diagram**

**Screening**

**Included**

**Eligibility**

**Identification**

Records identified through database searching
(n = 786 )

Additional records identified through other sources
(n = 0 )

Records after duplicates removed
(n = 768 )

Records screened
(n =168 )

Records excluded
(n = 12 )

Full-text articles assessed for eligibility
(n = 156 )

Full-text articles excluded, with reasons
(n =77 )

Studies included in qualitative synthesis
(n = 79 )

Studies included in quantitative synthesis (meta-analysis)
(n = 74 )
